# Murine and Bovine γδ T Cells Enhance Innate Immunity against *Brucella abortus* Infections

**DOI:** 10.1371/journal.pone.0021978

**Published:** 2011-07-12

**Authors:** Jerod A. Skyberg, Theresa Thornburg, MaryClare Rollins, Eduardo Huarte, Mark A. Jutila, David W. Pascual

**Affiliations:** Department of Immunology and Infectious Diseases, Montana State University, Bozeman, Montana, United States of America; Ohio State University Medical Center, United States of America

## Abstract

γδ T cells have been postulated to act as a first line of defense against infectious agents, particularly intracellular pathogens, representing an important link between the innate and adaptive immune responses. Human γδ T cells expand in the blood of brucellosis patients and are active against *Brucella in vitro*. However, the role of γδ T cells *in vivo* during experimental brucellosis has not been studied. Here we report TCRδ^−/−^ mice are more susceptible to *B. abortus* infection than C57BL/6 mice at one week post-infection as measured by splenic colonization and splenomegaly. An increase in TCRγδ cells was observed in the spleens of *B. abortus*-infected C57BL/6 mice, which peaked at two weeks post-infection and occurred concomitantly with diminished brucellae. γδ T cells were the major source of IL-17 following infection and also produced IFN-γ. Depletion of γδ T cells from C57BL/6, IL-17Rα^−/−^, and GMCSF^−/−^ mice enhanced susceptibility to *B. abortus* infection although this susceptibility was unaltered in the mutant mice; however, when γδ T cells were depleted from IFN-γ^−/−^ mice, enhanced susceptibility was observed. Neutralization of γδ T cells in the absence of TNF-α did not further impair immunity. In the absence of TNF-α or γδ T cells, *B. abortus*-infected mice showed enhanced IFN-γ, suggesting that they augmented production to compensate for the loss of γδ T cells and/or TNF-α. While the protective role of γδ T cells was TNF-α-dependent, γδ T cells were not the major source of TNF-α and activation of γδ T cells following *B. abortus* infection was TNF-α-independent. Additionally, bovine TCRγδ cells were found to respond rapidly to *B. abortus* infection upon co-culture with autologous macrophages and could impair the intramacrophage replication of *B. abortus* via IFN-γ. Collectively, these results demonstrate γδ T cells are important for early protection to *B. abortus* infections.

## Introduction

Brucellosis is a widespread and economically important agricultural and human disease in many regions of the world. The etiologic agent of brucellosis in cattle is *Brucella abortus*, a Gram-negative, facultative, intracellular pathogen [1]. The pathological manifestations of brucellosis are diverse, depending upon host, and in humans include arthritis, endocarditis, and meningitis, while animal brucellosis is characterized by spontaneous abortions [2]. Although use of the *B. abortus* vaccine strains S19 and RB51 reduces disease incidence and prevents *B. abortus*-induced abortions, these vaccines are less than ideal because of their limited efficacy and potential to cause disease in humans [3], [4]. Consequently, the need exists for improved animal protective measures that will combine safety and efficacy to all species at risk, including domestic herds and wildlife [5]. While much emphasis has been placed on identifying new vaccine candidates, relatively little attention has been paid to the effective coordination of the innate immune response by the host. Moreover, the need exists to determine protective innate immune responses against brucellosis to lessen the progression of infection and to allow a protective, adaptive immune response to develop [6].

γδ T cells constitute a small percentage of circulating T cells in adult humans and in mice, but comprise a majority of the intraepithelial lymphocyte population in the gut and in other epithelial mucosa [7]. Moreover, γδ T cells are a major subset in adult ruminants and make up a majority (up to 70%) of circulating lymphocytes in neonatal calves [8]. γδ T cells are the first to develop and are recruited and expand in response to various infections in humans, rodents, and other animals [7].

Studies conducted with TCRδ^−/−^ mice demonstrate γδ T cells can play a crucial role in protection from bacterial infection [9]–[12], and it has been proposed that mice lacking γδ T cells have an altered or impaired response to Gram-negative bacteremia [10]. Different mechanisms have been proposed for the protection conferred by γδ T cells in murine models. Moore *et al.* [10] postulates decreased levels of IFN-γ and TNF-α observed in *Klebsiella*-infected TCRδ^−/−^ mice results in enhanced susceptibility to infection. However, in studies with *Nocardia*-infected mice, it is shown that TCRδ^−/−^ mice are unable to effectively recruit inflammatory cells, leading to increased susceptibility to infection (and death) [9]. γδ T cells have also been shown to be a major producer of IL-17 in response to infection with intracellular bacteria [13], [14].

In humans, Vγ9δ2 T cells represent the major subtype of γδ T cells in blood, but make up only 1–5% of all circulating peripheral T cells [15]. However, the number of Vγ9δ2 T cells can dramatically increase in early response to infection by a number of intracellular pathogens, including *Brucella* [16]. Also, *B. suis* is shown to produce soluble factors that can directly activate Vγ9δ2 T cells to produce IFN-γ and TNF-α [17]. *In vitro*, Vγ9δ2 T cells exhibit strong cytolytic activity against *Brucella*-infected cells and are able to impair intracellular growth of *B. suis* in autologous macrophages [17]. Inhibition of bacterial multiplication is partially attributed to IFN-γ and TNF-α production by Vγ9δ2 T cells, while granule exocytosis is a mechanism used by Vγ9δ2 T cells to reduce the intracellular numbers of *B. suis* by lysing infected macrophages [15]. In addition, Vγ9δ2 T cells are shown to play a part in innate immunity to *Brucella* by releasing LL-37 as an antimicrobial defense mechanism [18]. While the effects of human Vγ9δ2 T cells against *Brucella* have been studied *in vitro*, this subset of γδ T cells is absent in nonhuman primates [15]. In addition, the role of γδ T cells in response to *Brucella* has not been studied *in vivo* in an animal model [6]. Here we demonstrate γδ T cells can be protective against *B. abortus* infection in both murine and bovine models.

## Results

### TCRδ^−/−^ mice have impaired innate immunity to *B. abortus* infection

**Figure 1:**
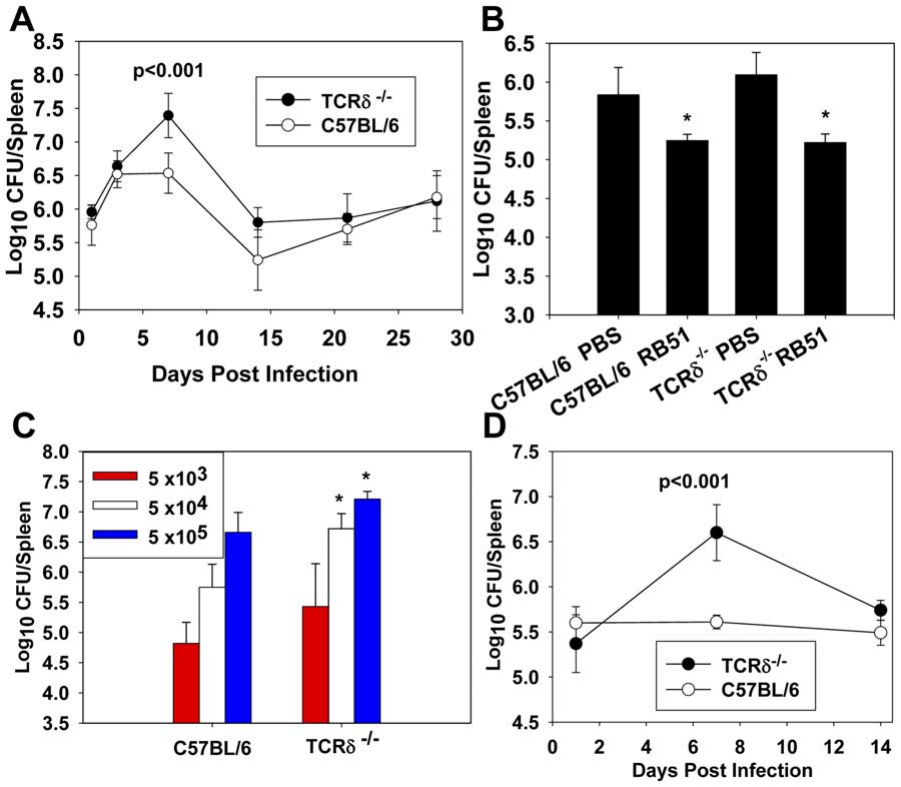
γδ T cells are required for innate, but not acquired, immunity to *B. abortus* infection. **A.** TCRδ^−/−^ and C57BL/6 mice were infected with 5×10^5^ CFUs of *B. abortus* 2308 i.p and splenic colonization was determined 1, 3, 7, 14, 21, and 28 days post-infection. The mean ± SEM of three mice/time point/group, except day 7, which contains 10 mice/group, is shown. **B.** TCRδ^−/−^ and C57BL/6 mice were dosed i.p. with sterile PBS or 1×10^8^ CFUs of *B. abortus* RB51 vaccine. Mice were challenged 8 wks post-immunization with 5×10^5^ CFUs of *B. abortus* 2308, and splenic colonization was determined four wks post-infection. The mean ± SD of 3–4 mice/group is shown; * P<0.05 versus unvaccinated mice of the same genotype. **C.** C57BL/6 and TCRδ^−/−^ mice were infected with 5×10^3^, 5×10^4^, or 5×10^5^ CFUs of *B. abortus* 2308 i.p., and splenic CFUs were determined at seven days post-infection. The mean ± SD of 4–5 mice/group is shown; * P<0.05 versus wild-type mice infected with the same dose of *B. abortus*. **D.** TCRδ^−/−^ and C57BL/6 mice were infected with 5×10^4^ CFUs of *B. abortus* 2308, and splenic colonization was determined 1, 7, and 14 days post-infection. The mean ± SEM of 4 mice/group except day 7 contains 13 mice/group is shown; * P<0.05 versus wild-type mice infected with *B. abortus*.

To assess the role of γδ T cells in protection against brucellosis, TCRδ^−/−^ and wild-type (wt) C57BL/6 mice were infected with *B. abortus*, and at select time points (1, 3, 7, 14, 21, and 28 days post-infection), the extent of splenic colonization was determined (Figure 1A). At one week post-infection, TCRδ^−/−^ spleens contained ∼ten-fold more *Brucella* than spleens from wt mice, and by two weeks post-infection, no significant differences in colonization were observed between TCRδ^−/−^ and wt mice. To determine if the protective role of γδ T cells was limited to innate immunity, wt and TCRδ^−/−^ mice were immunized with RB51 and eight weeks later challenged with *B. abortus* 2308. Four weeks after challenge, vaccination efficacy was assessed. RB51 was found to be equally protective in wt and TCRδ^−/−^ mice (Figure 1B), indicating the protective function of γδ T cells against *B. abortus* must be limited to early infection and most likely to innate immunity. In an effort to determine if the enhanced susceptibility of TCRδ^−/−^ mice to infection was dose-dependent, both wt and TCRδ^−/−^ mice were infected with varying doses, 5×10^3^, 5×10^4^, or 5×10^5^ CFUs of *B. abortus*, and splenic colonization was assessed one week post-infection (Figure 1C). TCRδ^−/−^ mice infected with either 5×10^4^ or 5×10^5^ CFUs of *B. abortus* were more susceptible to infection than wt mice (Figure 1C). The kinetics of infection and susceptibility following infection with 5×10^4^ CFUs are similar (Figure 1D) to that found with 5×10^5^ CFUs of *B. abortus* (Figure 1A).

### Splenic γδ T cells proliferate in response to *B. abortus* infection

To determine if γδ T cells are induced subsequent *B. abortus* infection, C57BL/6 mice were infected with 5×10^4^ CFUs of *B. abortus*, and the relative proportions of TCRγδ^+^, TCRαβ^+^, CD4^+^, and CD8^+^ T cells were determined at varying time points post-infection when compared to splenic T cells of naïve mice. Approximately a 3-fold increase in the proportion of γδ T cells was observed in the spleens of *B. abortus*-infected C57BL/6 mice, which peaked at two weeks post-infection and occurred concomitantly with diminished brucellae (Table 1; Figure 1A and D). As the total number of splenic mononuclear cells increased dramatically during infection, the increase in the proportion of γδ T cells observed was due to an increase in the absolute number of γδ T cells, rather than a reduction in the number of other lymphocyte subsets. The proportion of CD4^+^ T cells also appeared to decrease by day 28 following infection, which could be due to an increase in the proportion of B cells, a phenomenon that has been observed in other murine models in which mice are infected with intracellular bacteria, such as *Mycobacterium* [19].

**Table 1:** Percentages of splenic T cell subsets and splenic weights following *B. abortus* infection^a^.

	Uninfected	3 days PI	7 days PI	14 days PI	21 days PI	28 days PI
**TCRδ^+^**	0.52 (0.056)	0.73 (0.136)	0.50 (0.054)	1.84 (0.13)*	1.31 (0.031)*	0.80 (0.070)*
**TCRβ^+^**	37.50 (1.75)	34.73 (0.76)	38.05 (1.57)	38.80 (1.82)	26.33 (3.12)	27.29 (3.03)*
**CD4^+^**	27.20 (1.76)	22.23 (1.41)	24.14 (1.53)	27.40 (3.19)	16.67 (3.33)	16.77 (1.39)*
**CD8^+^**	10.17 (1.48)	9.34 (0.66)	11.40 (0.83)	9.50 (1.53)	10.77 (0.84)	7.85(1.36)
**Lymphocytes (×10^6^)**	77.3 (0.98)	ND	194.1 (3.53)*	357.5 (6.52)*	ND	ND
**Spleen weight (mg)**	75.3 (6.4)	86.0 (2.1)	170.0 (14.1)*	284 (37.3)*	275.3 (26.6)*	280.0 (27.7)*

a Spleen cells from *Brucella*-infected and naïve C57BL/6 mice were stained for FACS analysis using conventional methods. Total splenic lymphocyte number and spleen weights are shown. At least three mice were used per time point, while the 14 day time point represented six mice from two independent experiments. Standard error is shown in parentheses. ND = not determined.

*p<0.05 as compared to uninfected.

### γδ T cells from *B. abortus* infected mice produce IL-17 and IFN-γ

**Figure 2:**
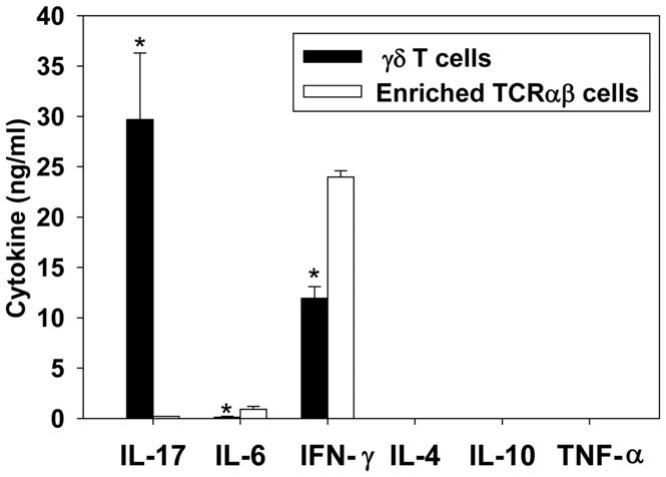
γδ T cells are the primary source of IL-17 during *B. abortus* infection. C57BL/6 mice were infected i.p. with 5×10^4^ CFUs of *B. abortus* 2308, and two weeks later γδ T cells (>95% purity) and an enriched TCRαβ (∼55% CD4^+^, 25% CD8^+^) cell fraction were isolated from the spleens of infected mice. Cells were stimulated with 500 ng/ml ionomycin and 50 ng/ml PMA for three days, and cell-free supernatants from triplicate wells were assayed for cytokine production via ELISA. The mean ± SD is shown; * P<0.05 versus the enriched TCRαβ cells. Results are representative of two independent experiments.

Since γδ T cells are enhanced by *B. abortus* infection, we queried which cytokines are produced. γδ T cells (>95% purity) were purified from C57BL/6 mice infected 14 days earlier. This time point was selected since it was the peak of splenic γδ T cell induction. For comparison, total T cells (neutralized of γδ T cells) were also assayed for cytokine production in parallel with the purified γδ T cells. Both T cell subsets were stimulated with ionomycin and PMA for three days (Figure 2). γδ T cells were found to be the major producer of IL-17 during infection and were also found to produce IFN-γ. IL-17 production by purified γδ T cells from *B. abortus*-infected mice was also found to be induced by TCRγδ stimulation (data not shown). Minimal IL-17 was produced by the enriched TCRαβ cell fraction; however, this fraction was found to contain elevated levels of IFN-γ and IL-6 relative to γδ T cells. IL-4, IL-10, and TNF-α were not detected in any cell culture supernatants.

### γδ T cells do not require IL-17Rα, IFN-γ, or GM-CSF to confer protection against systemic *B. abortus* infection

**Figure 3:**
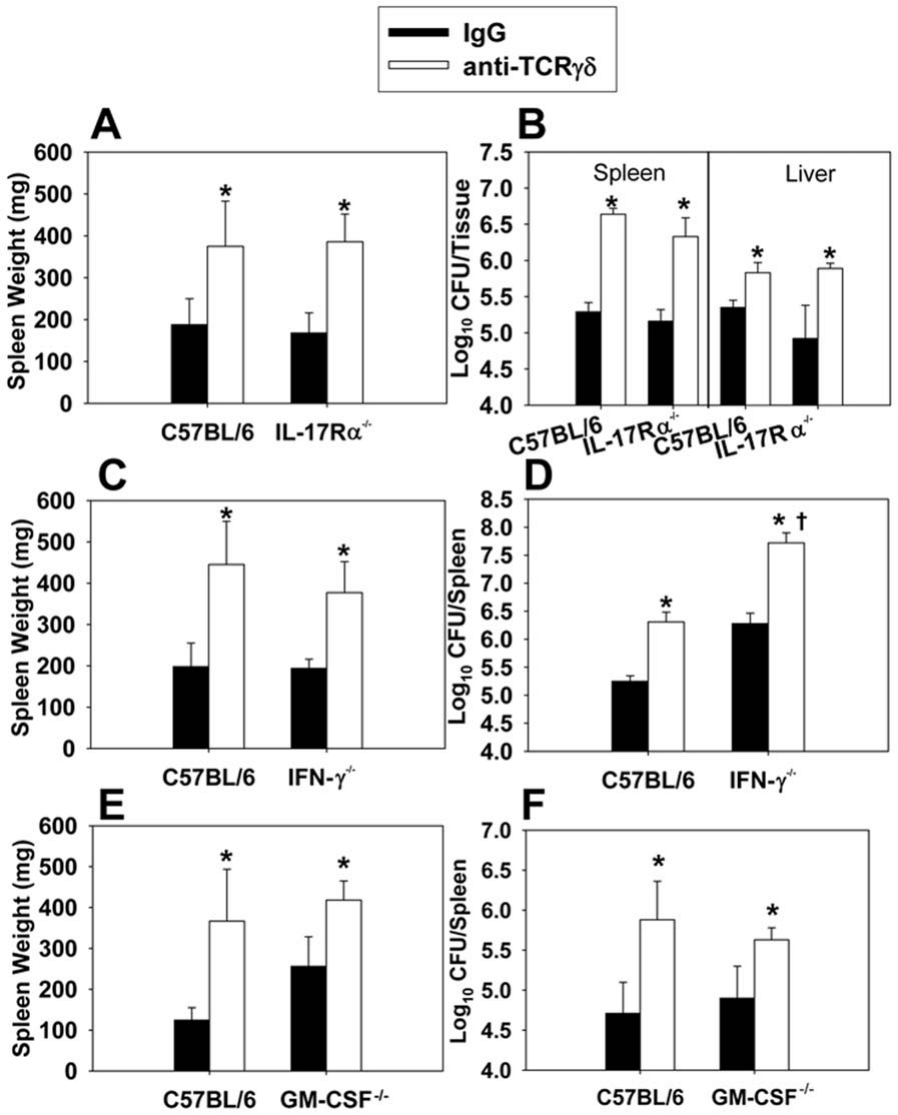
γδ T cells do not require IL-17Rα, IFN-γ, or GM-CSF to mediate protection to *B. abortus* infection. **A.–F.** C57BL/6 or **A.** and **B.** IL-17R**α** ^−/−^, **C.** and **D.** IFN-**γ** ^−/−^, and **E.** and **F.** GM-CSF^−/−^ mice (5–6 per group) were treated with anti-TCR γδ mAb or hamster IgG on day -1 and day 3 post-infection with 5×10^4^ CFUs of *B. abortus* 2308. Mice were sacrificed seven days after infection. **A.**, **C.**, and **E.** for splenic weights and **B.**, **D.**, and **F.** for tissue colonization were determined; *P<0.05 versus mice of the same genotype treated with hamster IgG; ^†^ P<0.05 versus anti-TCRγδ-treated C57BL/6 mice.

Since γδ T cells were found to be the main source of IL-17 during infection, we sought to determine if the protection conferred by γδ T cells was IL-17-dependent. Wt and IL-17 receptor deficient (IL-17Rα^−/−^) mice were neutralized of γδ T cells via neutralizing mAb (control mice received normal hamster IgG) and were then infected with *B. abortus*. Neutralization of γδ T cells enhanced the susceptibility of both wt and IL-17Rα^−/−^ mice (Figure 3A and B), and IL-17 receptor deficiency did not impact splenomegaly (Figure 3A), nor tissue colonization by *B. abortus* (Figure 3B) at one week post-infection relative to infected wt mice. Thus, these data show that the protective effect by γδ T cells during infection is independent of IL-17. Intracellular cytokine staining revealed that while γδ T cells were the main source of IL-17 during infection, the proportion of cells producing IL-17 was actually diminished by *B. abortus* infection (Figure S1A), which may explain while IL-17Ra was dispensable for protection.

IFN-γ is required for immunity to experimental brucellosis [20]–[22] and also is produced by γδ T cells from *B. abortus*-infected mice. Therefore, wt and IFN-γ^−/−^ mice (B6 background) were neutralized of γδ T cells via neutralizing mAb (control mice received normal hamster IgG) and were then infected with *B. abortus* (Figure 3C and D). Deficiency of either IFN-γ or γδ T cells enhanced susceptibility to colonization by ∼10-fold at one-week post-infection, while mice deficient in both IFN-γ and γδ T cells possessed >300-fold more viable *B. abortus* in their spleens than did wt mice (Figure 3D). IFN-γ^−/−^ mice neutralized of γδ T cells prior to infection with *B. abortus* displayed ruffled fur and a hunched posture (data not shown), while no clinical symptoms of disease were observed in any other treatment group. Thus, the protection conferred by γδ T cells was independent of IFN-γ. In addition, γδ T cell deficiency in wt mice resulted in enhanced splenomegaly and brucellar colonization, while splenomegaly in IFN-γ^−/−^ mice was similar to that observed wt mice (Figure 3C and D). GM-CSF has been found by others to be required for protection to intracellular bacterial pathogens [23], [24]; however, GM-CSF was not important for protection against *B. abortus* and was dispensable for γδ T cell-mediated protection against colonization and splenomegaly at one week post-infection (Figure 3E and F).

### γδ T cell-mediated protection against B. abortus infection is dependent on TNF-α

**Figure 4:**
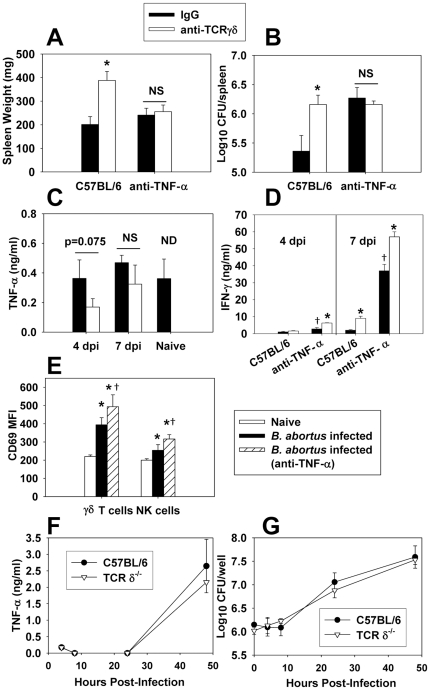
γδ T cells require TNF-α to protect against *B. abortus* infection. C57BL/6 mice treated with anti-TCRγδ mAb or hamster IgG on day -1 and day 3 post-infection with 5×10^4^ CFUs of *B. abortus* 2308. Some mice were also neutralized of their TNF-α on days -1 and 3. Seven days after infection, **A.** splenic weights and **B.** extent of brucellae colonization were determined. The mean ± SEM of 10 mice/group is depicted; *P<0.05 versus hamster IgG-treated C57BL/6 mice. Results are from two independent experiments. **C.** and **D.** Splenocytes (5×10^6^/ml) from 4 or 7 day *B. abortus*-infected mice depleted or not of γδ T cells and/or TNF-α or splenocytes from naïve C57BL/6 mice were left unstimulated and cultured for 3 days at 37°C/5%CO_2_; supernatants were harvested for **C.** TNF-α- or **D.** IFN-γ- specific ELISA. The mean ± SD of triplicate wells is shown. The results from 7 dpi are representative of two independent experiments. NS = not significant. ND = cytokine production from uninfected mice neutralized of γδ T cells was not determined. * P<0.05 as compared to mice not depleted of γδ T cells within the same TNF-α treatment group at the same time point. ^†^ P<0.05 as compared to C57BL/6 mice treated with IgG only at the same time point. **E.** The median fluorescence intensity (MFI) of CD69 expression by splenic NK and γδ T cells as measured by flow cytometry is shown for naïve C57BL/6 and *B. abortus*-infected (after 4 days of infection with 5×10^4^ CFUs of strain 2308) C57BL/6 mice. Data depict the mean ± SD from 5 mice/group; *P<0.05 versus naïve mice and ^†^ P<0.05 versus *B. abortus*-infected mice not neutralized of their TNF-α. **F.** and **G.** Peritoneal macrophages from C57BL/6 and TCRδ^−/−^ mice were infected with *B. abortus* (30 bacteria∶1 macrophage), and **F.** TNF-α levels in supernatants and **G.** intracellular colonization were measured. Data represent the mean ± SD of triplicate wells/group.

While γδ T cells were found not to be a major producer of TNF-α (Figure 2), others have shown TNF-α is required for protection to *B. abortus* [25]. Thus, mice were neutralized of γδ T cells, TNF-α, or both, prior to infection with *B. abortus*. Neutralization of either TNF-α or γδ T cells from wt mice enhanced susceptibility to *B. abortus* colonization one week after infection (Figure 4A and B). However, *in vivo* neutralization of TNF-α did not exacerbate susceptibility to colonization or splenomegaly in mice neutralized of γδ T cells (Figure 4B), indicating TNF-α is required for γδ T cell-mediated protection against *B. abortus*. Depletion of γδ T cells did not appear to affect TNF-α production by splenocytes from *B. abortus*-infected mice at 4 or 7 days post-infection (Figure 4C), and stimulation of splenocytes with plate-bound anti- γδ TCR mAb did not enhance TNF-α production (data not shown). Also, TNF-α production by splenocytes from naïve and *B. abortus*-infected mice was similar (Figure 4C), and intracellular TNF-α levels in splenocytes from both *B. abortus*-infected and naïve mice were similar as measured by flow cytometry (data not shown). However, increased IFN-γ production by splenocytes from *B. abortus*-infected mice was observed in mice depleted of TNF-α and/or γδ T cells (Figure 4D), suggesting possible compensation by IFN-γ. Others have shown that TNF-α preferentially activates γδ T cells as measured by up-regulation of surface CD69 expression [26]. While *B. abortus* infection did lead to activation of γδ T cells (and NK cells to a lesser extent), this effect was independent of TNF-α at 4 and 7 days post-infection, as mice neutralized of TNF-α *in vivo* still displayed enhanced CD69 expression by their γδ T cells and, in fact, more so than mice simply subjected to *B. abortus* infection (Figure 4E and data not shown). In addition, it has been shown that macrophages from TCRδ^−/−^ mice display impaired TNF-α production when stimulated *in vitro* with LPS [27]. However, no differences in the ability of wt or TCRδ^−/−^ peritoneal macrophages to produce TNF-α or control brucellae infection was evident (Figure 4F and G).

### Bovine γδ T cells can impair the intramacrophage growth of *B. abortus* in autologous macrophages

**Figure 5:**
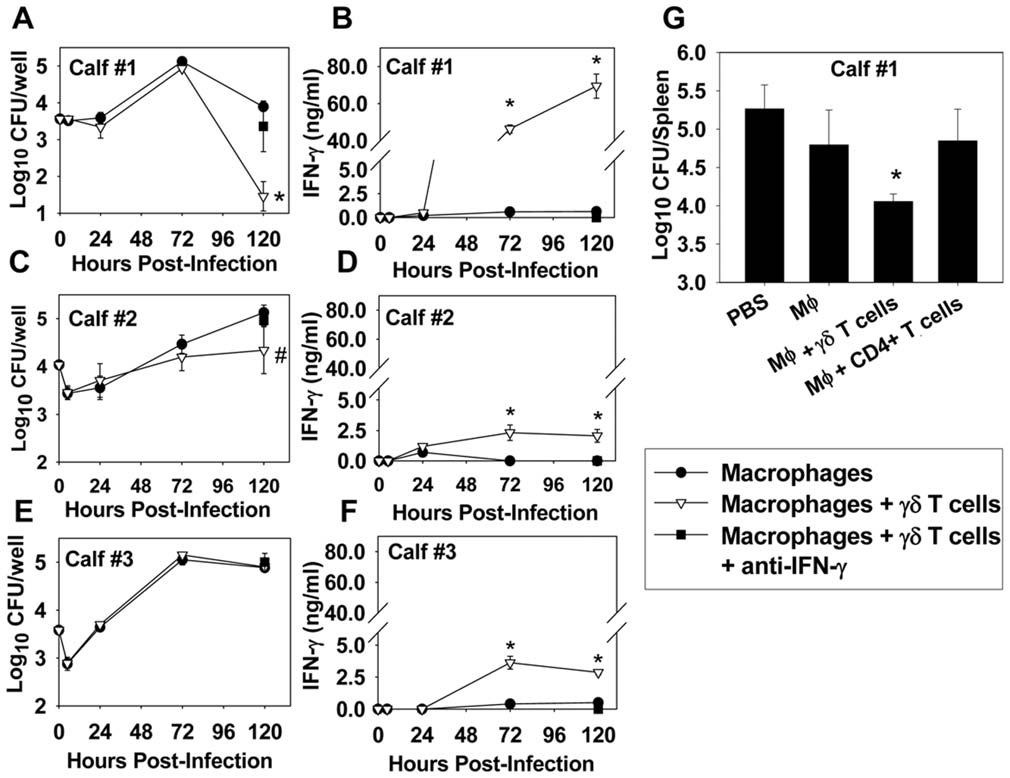
Bovine γδ T cells impair *B. abortus* replication in autologous macrophages via IFN-γ. **A.–F.** Bovine macrophages were infected with *B. abortus* (30 bacteria∶1 macrophage) and then fresh media or media containing autologous γδ T cells were added to infected macrophages. **A.**, **C.**, **E.** Macrophage colonization (triplicate wells/treatment) was monitored over time. *P<0.05 versus colonization by cultured macrophages only from the same animal. **B.**,**D.**,**F.** IFN-γ levels were determined in cell culture supernatants. At 72 and 120 h post-infection, macrophage plus γδ T cell co-cultures from each calf contained more IFN-γ than from macrophages cultured alone. **G.** NK1.1^+^ cell-depleted Rag-1^−/−^ mice (4–5 per group) received PBS, bovine macrophages (5×10^5^/mouse), bovine macrophages (5×10^5^/mouse) plus autologous γδ T cells (1×10^7^/mouse), or bovine macrophages plus autologous CD4^+^ T cells (1×10^7^/mouse) i.p. one day prior to infection with 1×10^4^ CFUs of *B. abortus*. All cells were derived from Calf #1. On day 7, splenic colonization was determined. Depicted is the mean ± SD; *P<0.05 versus mice given PBS or macrophages only.

In order to corroborate our findings with murine γδ T cells in a bovine model, a co-culture system was used in which bovine macrophages were infected with *B. abortus*, and then fresh media containing either media only, or media including autologous γδ T cells were added to the macrophage-containing wells. Intracellular bacterial burden was then determined at several time points post-infection. We found that at 5 days post-infection, bovine γδ T cells could augment the clearance of *B. abortus* in autologous macrophages. This effect varied depending on the donor of leukocytes, as γδ T cells from Calf #1 were protective (Figure 5A); however, γδ T cells from Calf #2 (Figure 5C) and Calf #3 were not as effective (Figure 5E). Protection appeared to correlate with IFN-γ production, and the addition of γδ T cells to macrophage containing wells from all donors resulted in an increase in IFN-γ concentration (Figure 5B, D, and F); however, the addition γδ T cells from Calf #1 resulted in the greatest increase in IFN-γ production (Figure 5B). Neutralization of IFN-γ *in vitro* also abrogated the protective effect γδ T cells from Calf #1, indicating an IFN-γ-dependent mechanism. To assess whether γδ T cells could protect against *B. abortus* in an *in vivo* adoptive transfer model, Rag-1^−/−^ mice were depleted of NK cells and reconstituted with bovine macrophages, macrophages plus autologous γδ T cells, or macrophages plus autologous CD4^+^ T cells prior to infection with *B. abortus* (leukocytes for adoptive transfer were all derived from Calf #1). When mice were sacrificed seven days later, adoptive transfer of macrophages with γδ T cells, but not transfer of macrophages only, or macrophages with CD4^+^ T cells, resulted in a significant reduction in splenic colonization by brucellae (Figure 5G).

### Bovine γδ T cells respond rapidly to *B. abortus* infection and alter the transcriptional profile of autologous macrophages

**Figure 6:**
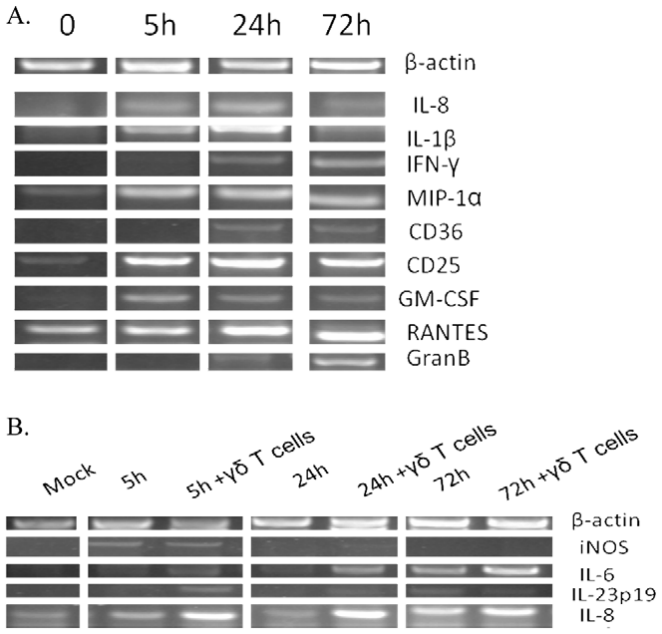
Transcriptional response of bovine γδ T cells to infection. **A.** Total RNA was extracted from resting γδ T cells, or γδ T cells co-cultured with infected autologous macrophages for 5, 24, or 72 h. **B.** RNA was also isolated from mock- or *B. abortus*-infected macrophages cultured with or without γδ T cells after removal of γδ T cells and repeated washing of adherent macrophages. cDNA was synthesized, and RT-PCR was conducted for several immune-related genes and β-actin Results are representative of two independent experiments with Calf #1.

**Figure 7:**
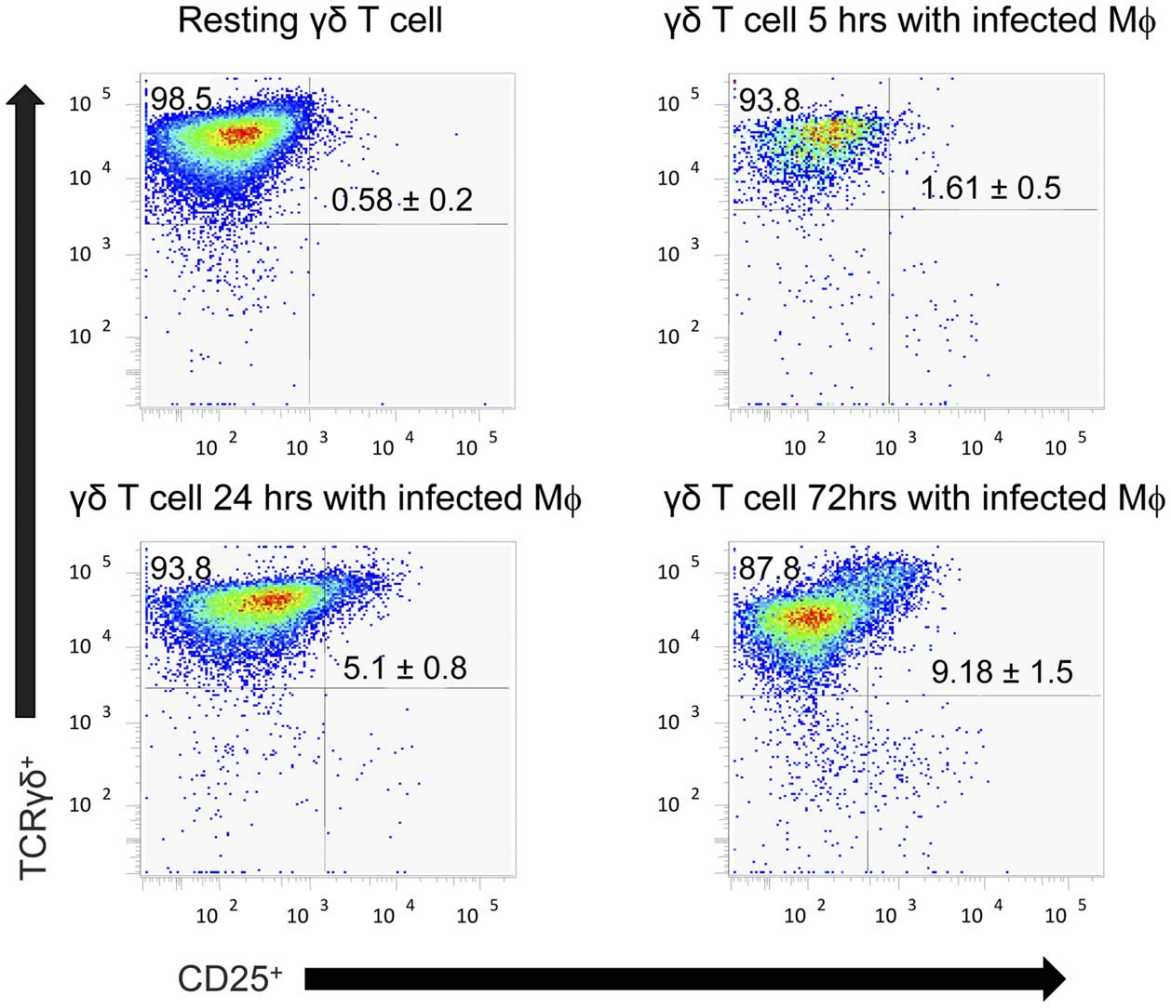
Bovine γδ T cells upregulate surface CD25 expression following infection of autologous macrophages with *B. abortus*. Surface CD25 expression was measured on naïve γδ T cells along with γδ T cells co-cultured with *B. abortus*-infected autologous macrophages for 5, 24, or 72 h. Data depict the mean ± S.D. of triplicate measurements/group.

To determine the transcriptional responses of protective bovine γδ T cells during infection at several time points, non-adherent γδ T cells were aspirated from macrophage-containing wells, and RNA was extracted. Following aspiration of γδ T cells, the remaining (adherent) macrophages were washed repeatedly to remove any remaining non-adherent cells. This process allowed determination of the transcriptional responses by both cell populations via RT-PCR. After 5 h of co-culture with autologous *B. abortus*-infected bovine macrophages, γδ T cell mRNA transcripts for IL-8, IL-1β, GM-CSF, MIP-1α, and CD25 were up-regulated (Figure 6A). A slight up-regulation of IL-17 after 5 and 24 h of co-culture was also observed in γδ T cells (data not shown). Later in infection, particularly after 72 h of co-culture, γδ T cells produced markedly stronger granzyme B, RANTES, IFN-γ, and CD36 transcripts (Figure 6A). To corroborate the transcriptional analysis at the protein level, surface expression for CD25 (IL-2Rα) by γδ T cells was assayed via flow cytometry. Co-culture of γδ T cells with *B. abortus*-infected macrophages enhanced CD25 expression by γδ T cells within 5 h, and by 72 h, the percentage of γδ T cells expressing CD25 had increased >15 fold (Figure 7). The presence of γδ T cells also altered the transcriptional profile of macrophages in the co-culture. Bovine macrophages produced elevated IL-6 mRNAs following infection with *B. abortus*; however, this response was accelerated and amplified by γδ T cells (Figure 6B). γδ T cells also enhanced the expression of IL-8 and IL-23p19 by macrophages, particularly, early in infection. IL-8 was also detected within 5 h of γδ T cells/macrophage culture, but was not detected in wells containing macrophages only (Figure S2). iNOS mRNA expression was only detected at 5 h post-infection, a response unaltered by the presence of γδ T cells. Significant changes in mRNA levels for IL-4, IL-10, IL-12p35, IL-12p40, TNF-α, FoxP3, and TGF-β mRNA from γδ T cells or macrophages were not observed under the conditions tested (data not shown).

## Discussion

With over 500,000 new human cases a year [28], brucellosis is the most common zoonotic infection in the world [29]. The pathological manifestations of brucellosis are diverse and include arthritis, endocarditis, and meningitis in humans, while animal brucellosis is characterized by spontaneous abortion [2]. While much emphasis has been placed on identifying new vaccine candidates for human and animal brucellosis, relatively little attention has been paid to effective coordination of the innate immune response by the host. γδ T cells represent a small percentage of circulating T cells in adult humans and mice, [7]; however, γδ T cells are a major lymphocyte subset in adult ruminants and make up a majority (up to 70%) of circulating lymphocytes in neonatal calves [8]. γδ T cells play an active role in the regulation and resolution of pathogen induced immune responses [30]; however, different subsets of γδ T cells may have different functions. Transcriptional analyses of bovine γδ T cells suggest that while CD8^−^ γδ T cells are activated, proliferative, and inflammatory, the CD8^+^ subset suppressed genes are consistent with quiescence trafficking to the mucosa and immune suppression [7]. While human Vγ9δ2 T cells exhibit strong cytolytic activity against *Brucella*-infected cells and are able to impair intracellular growth of *B. suis* in autologous macrophages [17], this subset of γδ T cells is absent in nonhuman primates, and the role of γδ T cells in an *in vivo* model of brucellosis has not been determined.

Here we report mice deficient in γδ T cells had impaired innate immunity to *B. abortus*. The protective role of γδ T cells during infection appeared to be temporal, as TCRδ^−/−^ mice were more susceptible to *B. abortus* colonization at 7 days post-infection; however, the absence of γδ T cells did not impair immunity, particularly, at time points after 2 wks post-infection. Vaccination of mice with RB51 before challenge with wild-type *B. abortus* 2308 also resulted in reduced colonization in wt and TCRδ^−/−^ mice, suggesting that the protective function of γδ T cells may be limited to innate immunity. In addition, the temporal protection conferred by γδ T cells correlates with the theory that γδ T cells may be a link between innate and adaptive immunity [31]. Studies in which γδ T cells are protective against the organism *Listeria monocytogenes* [32] have found the magnitude and timing of the γδ T cell response are related to the infectious dose of bacteria used, with γδ T cell expansion being quicker and of a larger magnitude in affected organs following a higher dose of bacteria [33]. Therefore, as our initial studies used a high dose of *B. abortus* (5×10^5^ CFUs), we queried whether the protection mediated by γδ T cells in our model was dose-dependent by infecting wt and TCRδ^−/−^ mice with three doses of *B. abortus* (5×10^3^, 5×10^4^, and 5×10^5^ CFUs). Protection against colonization conferred by γδ T cells at 7 days post-infection appeared to be similar, regardless of the dose of infection, and the difference in the kinetics of clearance of *B. abortus* between wt and TCRδ^−/−^ mice was also similar, regardless of whether 5×10^4^ or 5×10^5^ CFUs were used as an infectious dose. Notably, regardless of whether TCRδ^−/−^ mice or wt mice neutralized of γδ T cells via mAb (clone UC7-13D5) treatment was used, either model showed similar impairment of immunity to *B. abortus*, indicating that the effect of γδ T cells is not due to a developmental defect in TCRδ^−/−^ mice. A recent study has shown that treatment of mice with mAb against the TCRγδ (such as UC7-13D5) may not actually deplete cells, but rather block TCRγδ signaling [34], which according to the results presented here would indicate the protective effect of murine γδ T cells against *B. abortus* requires TCRγδ-specific signaling.

To determine if γδ T cells expand in response to infection, the lymphocyte composition of spleens from *B. abortus*-infected C57BL/6 mice was characterized at several time points post-infection. At two weeks post-infection, the proportion of γδ T cells amongst lymphocytes in the spleens from infected mice increased nearly three-fold. The total number of lymphocytes in spleens increased following *B. abortus* infection, indicating the enhanced percentage of γδ T cells was due to an increase in the total number of γδ T cells rather than a reduction of other T cell subsets. The finding that γδ T cells expanded or were recruited to the spleens of infected mice lent further support to the importance of γδ T cells in resolving infection by *Brucella*. The observed spike in splenic γδ T cells occurring after the peak of *Brucella* infection was similar to what has been found in mice infected with *Nocardia asteroides* or *Mycobacterium bovis* BCG [9], [35].

Different mechanisms have been proposed for the protection conferred by γδ T cells in murine models. *Klebsiella*-infected TCRδ^−/−^ mice are found to have decreased levels of IFN-γ and TNF-α, which has been postulated to result in enhanced susceptibility to infection [10]. γδ T cells are the main producer of IL-17 in naïve mice [14], and IL-17 production by γδ T cells has been shown to be augmented in response to infection [11], [13], [14]. In addition, the protection conferred by γδ T cells against infection with *L. monocytogenes*, *E. coli*, and *F. tularensis* LVS has been shown to be dependent on IL-17 [11], [13], [36]. Therefore, the γδ T cell cytokine profile was assessed following infection with *B. abortus* to ascertain the mechanism by which these cells confer protection. Similar to what others have found, γδ T cells were the main producer of IL-17 in infected mice. Interestingly, IL-17Rα^−/−^ mice were not impaired in their ability to control *B. abortus* infection, and IL-17Rα signaling was not required for γδ T cell-mediated protection. Intracellular cytokine staining revealed that while γδ T cells were the major source of IL-17 in both naïve and *B. abortus*-infected mice, IL-17 production was not enhanced by *Brucella* infection(Figure S1A–B). This finding explains why IL-17Rα is dispensable for protection against systemic challenge with *B. abortus*. While IFN-γ was produced by purified γδ T cells from *B. abortus*-infected mice, production of IFN-γ by an enriched TCRαβ cell fraction was much more robust. *B. abortus*-induced IFN-γ production by CD4^+^ T cells was confirmed by intracellular cytokine staining (Figure S1A–B). Subsequent secretion analysis of γδ T cells sorted from naïve and *B. abortus* infected TCRα^−/−^ mice (which have a higher proportion of γδ T cells) revealed that *B. abortus* infection may actually suppress IL-17 and IFN-γ production by γδ T cells (Figure S1B). However, it is important to consider the function of γδ T cells may be different in TCRα^−/−^ mice than in wt mice, and we must not over-interpret these results. The protective effects of IFN-γ and γδ T cells against *B. abortus* were found to be independent of each other and, in fact, could be compensatory, as depletion of γδ T cells from IFN-γ^−/−^ mice resulted in a greater impairment of immunity to *B. abortus* than did depletion of γδ T cells from wt mice. Indeed, depletion of TNF-α and/or γδ T cells from *B. abortus*-infected mice was found to enhance IFN-γ production by splenocytes. This finding further suggested the host may enhance IFN-γ production to compensate for the loss of protection conferred by TNF-α and/or γδ T cells and may also indicate the protective effects of IFN-γ in C57BL/6 mice may be independent of TNF-α and/or γδ T cells.

γδ T cells were not found to be a major producer of TNF-α, and neutralization of γδ T cells from *B. abortus*-infected mice did not significantly reduce TNF-α production. Others have shown that macrophages from TCRδ^−/−^ mice display impaired TNF-α production upon *in vitro* stimulation [27], yet no differences in the ability of peritoneal macrophages from either wt or TCRδ^−/−^ mice to control brucellae infection or produce TNF-α were observed. However, TNF-α was required for γδ T cells to confer protection against *B. abortus* infection. While it has been shown that TNF-α preferentially activates γδ T cells as measured by up-regulation of surface CD69 expression [26], TNF-α neutralization had no effect upon CD69 expression by γδ T cells or NK cells in *B. abortus*-infected mice. TNF-α is also known to enhance T cell cytotoxicity [37]; thus, future studies will investigate whether γδ T cells mediate protection against *B. abortus* infection via TNF-α-dependent cytotoxic effects. Interestingly, *B. abortus* infection enhanced CD69 expression more robustly in γδ T cells than in NK cells, which may help explain work by others indicating that NK cells are dispensable for innate immunity to *B. abortus* [38].

In order to corroborate our murine findings using bovine lymphocytes, a co-culture system was adopted and purified bovine γδ T cells were cultured with autologous *B. abortus*-infected macrophages. Bovine γδ T cells could impair the intramacrophage replication of *B. abortus* by co-cultured macrophages, but this effect varied depending on the donor of γδ T cells, which was not entirely surprising, since cattle exhibit wide variability in their immune response to infection [39]. While the addition of γδ T cells from all donors to autologous *B. abortus*-infected macrophages resulted in enhanced IFN-γ production in cell culture supernatants, γδ T cells only conferred significant protection when high levels (>40 ng/ml) of IFN-γ were produced. Neutralization of IFN-γ *in vitro* abrogated the protective effect of bovine γδ T cells in our co-culture system, further demonstrating the requirement of IFN-γ for bovine γδ T cell-mediated protection. IFN-γ has been shown to enhance the resistance of bovine macrophages to bacterial infection by inducing apoptosis [40]; therefore, future studies will assess whether γδ T cells and IFN-γ affect bovine macrophage apoptosis during *B. abortus* infection.

Transcriptional analyses of bovine co-cultured with *Brucella*-infected macrophages suggest γδ T cells follow a “priming” model of activation [41]. Early in infection (∼5 h), IL-8, MIP-1α (CCL3), GM-CSF, IL-1β, IL-17, and CD25 mRNAs were upregulated by γδ T cells. At later time points after infection (∼72 h), γδ T cells enhanced expression of granzyme B, RANTES, and IFN-γ mRNAs. This biphasic immune response indicates that γδ T cells initially respond to infection by expressing signals that lead to direct enhancement of macrophage function, along with an enhanced responsiveness of the γδ T cells to further secondary signals, such as antigen or cytokines, while later in infection, γδ T cells express signals indicative of an “effector” state [41]. In addition, the presence of γδ T cells seemed to alter the early mRNA profile of infected macrophages as increased levels of IL-8, IL-6, and IL23p19 were observed in infected macrophages co-cultured with γδ T cells relative to infected macrophages alone. These cytokines are all considered part of the Th17 response [42], suggesting that the presence of γδ T cells may shift the subsequent immune response to infection. TNF-α mRNA was not found to be induced in bovine cells under the conditions tested, which is not surprising as others have found that intravenous or subcutaneous vaccination of cattle with *B. abortus* RB51 does not increase serum TNF-α levels [43].

The need for model systems to analyze the immune system and pathogenesis of disease in cattle is great since studies in cattle are difficult owing to the limited availability of genetically similar animals and the high costs involved in animal purchase and housing [44]. *In vivo* bovine studies with virulent *Brucella* species are particularly difficult to perform due to the limited availability of large animal BSL-3 facilities. Therefore, to determine if bovine γδ T cells that were protective in our co-culture system could protect in an *in vivo* model, Rag-1^−/−^ mice were depleted of NK cells and reconstituted with bovine macrophages alone, macrophages plus autologous γδ T cells, or macrophages plus autologous CD4^+^ T cells prior to infection with *B. abortus*. When mice were sacrificed seven days later, adoptive transfer of macrophages with γδ T cells, but not macrophages only nor macrophages with CD4^+^ T cells, resulted in a significant reduction in splenic colonization by *B. abortus*. These findings further demonstrate bovine γδ T cells can protect against *B. abortus* infection, and reconstitution of Rag-1^−/−^ mice with bovine cells is a viable method, which can be used to assay the role of bovine leukocytes during infection.

In this study, we showed that both murine and bovine γδ T cells rapidly responded and could provide protection against, infection with *B. abortus*. While murine and bovine γδ T cells utilized different mechanisms of protection to confer protection against *B. abortus*, γδ T cells from both species were found to produce IFN-γ, enhance surface expression of activation markers, and help coordinate the host cytokine response following *B. abortus* infection. Characterization of the transcriptional profile of bovine γδ T cells subsequent to infection revealed a biphasic immune response. Initially, bovine γδ T cells expressed signals leading to direct enhancement of macrophage function along with enhanced γδ T cells responsiveness to further secondary signals, while later in infection γδ T cells expressed signals indicative of an effector state. Collectively, these findings demonstrate γδ T cells are important for controlling *B. abortus* infection and provide insight into the response of both murine and bovine γδ T cells in response to infection. In addition, these results indicate immunotherapeutic strategies that target γδ T cells could be viable means to augment innate immunity against brucellosis.

## Materials and Methods

### Ethics Statement

All animal care and procedures were in accordance with institutional policies for animal health and well-being, and approved by MSU Institutional Animal Care and Use Committee under protocol 38.

### Mice

Breeder pairs of γδ T cell-deficient (TCRδ^−/−^), TCRαβ-deficient (TCRα^−/−^), T and B cell-deficient (Rag-1^−/−^), and GM-CSF (GM-CSF^−/−^) deficient mice on a C57BL/6 background were obtained from The Jackson Laboratory (Bar Harbor, ME), and breeder pairs IL-17Rα^−/−^ mice on B6 background were a gift from Amgen (Seattle, WA); mice were bred and maintained at the Montana State University Animal Resource Center (Bozeman, MT). C57BL/6 and IFN-γ^−/−^ mice (B6 background; Jackson Laboratory) were used at 7 to 11 weeks of age. All mice were maintained at Montana State University Animal Resource Center under pathogen-free conditions in individually ventilated cages under HEPA-filtered barrier conditions and were fed sterile food and water *ad libitum*. In some experiments, γδ T cells and/or TNF-α were neutralized *in vivo* via intraperitoneal (i.p.) administration of 0.5 mg of anti-γδ TCR mAb (clone UC7-13D5; BioXcell) and/or 0.5 mg of anti-TNF-α mAb (clone XT3.11; BioXcell) on days -1 and 3 post-infection. For challenge studies with *B. abortus* strain 2308, mice were maintained under similar isolation conditions, in our institutional ABSL-3 facilities. All animal care and procedures were in accordance with institutional policies for animal health and well-being.

### Bacterial Strains and Growth Conditions

*B. abortus* strain 2308 and the *B. abortus* vaccine strain RB51 were obtained from the National Veterinary Services Laboratory, USDA (Ames, IA). Bacteria were grown under aerobic conditions in potato infusion agar for 72 h (Difco Laboratories) at 37°C and 5% CO_2_. For inoculation, a colony was chosen and incubated overnight at 37°C with shaking in *Brucella* broth (Difco); the bacterial suspension was adjusted spectrophotometrically to an optical density at 600 nm corresponding to desired inoculum concentration. All experiments with live brucellae were performed in our institutional biosafety level 3 facilities.

### Tissue Colony Counts

At selected time points post-infection, spleens and livers were harvested for CFU determinations. Organs were dounce homogenized, and serial 10-fold dilutions in triplicate of homogenates in sterile water were grown on *Brucella* agar (BA). After incubation for 3 to 5 days at 37°C with 5% CO_2_, *Brucella* colonies were enumerated, and the number of CFU per tissue was calculated from the dilutions.

### FACS Analysis

Murine and bovine lymphocytes were stained for FACS analysis using conventional methods [45], [46]. Following the removal of erythrocytes, whole murine spleen cells were stained with fluorochrome-conjugated or biotinylated mAbs (Becton Dickinson or eBioscience): anti-CD4 (clone L34T4), anti-CD8 (clone 853-6.7), anti-TCRβ chain (clone H57-597), anti-B220 (clone RA3-6B2), anti-NK1.1 (clone PK136), anti-CD69 (clone H1.2F3), anti-CD3 (clone 17A2), or anti-TCRδ chain (clone GL3); cells were then fixed with 2% paraformaldehyde. Bovine lymphocytes were stained with anti-TCRδ chain (clone GD3.8) and anti-CD25 (clone LCTB2A), as described [47]. Stained lymphocytes were analyzed using a FACSCalibur, FACSCanto, or LSRII flow cytometer (BD Biosciences) and analyzed using FlowJo software (Tree Star). For intracellular staining, cells were isolated as described above and stimulated overnight with ionomycin (500 ng/ml)/phorbol 12-myristate 13-acetate (PMA; 50 ng/ml). For the last 3 h of culture, brefeldin A (10 µg/ml) was added to the cultures. Cells were then stained for cell surface markers as described above and fixed in paraformaldehyde. Cells were then permeabilized with 0.2% saponin prior to intracellular staining for IL-17A (clone TC11-18H10) and IFN-γ (clone XMG1.2). For determination of total lymphocytes, splenic mononuclear cells were isolated over a Lympholyte M gradient (Cedarlane Labs), and viable cells counted via trypan blue exclusion. The proportion of lymphocytes was confirmed via flow cytometry by gating on forward and side scatter.

### Isolation and Infection of Murine Peritoneal Macrophages

Peritoneal macrophages were isolated, as previously described [48], [49]. Briefly, mice were given a single i.p. injection of 1.0 ml of expired thioglycolate medium (Difco), and 3 days later, the peritoneum of each mouse was washed with RPMI 1640 (Gibco BRL-Life Technologies [Life Technologies] containing 2% fetal calf serum (Life Technologies) without antibiotics. Peritoneal cells were washed twice in the same medium without antibiotics and allowed to adhere overnight to 24 well microtiter plates. Macrophages (1×10^6^/ml) were infected with *B. abortus* (30 bacteria∶1 macrophage) for 1 hour at 37°C/5% CO_2_. Macrophages were then washed with PBS, fresh complete medium (CM): RPMI 1640 medium supplemented with 1 mM sodium pyruvate, 1 mM nonessential amino acids, penicillin/streptomycin (10 U/ml), and 10% FBS (Atlanta Biologicals) containing 50 µg/ml gentamicin (Sigma-Aldrich) were added, and cells were incubated for 30 min at 37°C/5% CO_2_. After washing twice, fresh CM containing gentamicin (2.5 µg/ml) was added, and cells were incubated 37C/5% CO_2_. At various time points post-infection, the wells were washed three times, macrophages were lysed with sterile water, and intracellular bacterial burden was determined by serial dilution of macrophage lysates on BA.

### Cytokine ELISAs

Spleens were aseptically removed from C57BL/6 mice at various time points after challenge with *B. abortus* 2308. For whole cell cultures, murine splenocytes (5×10^6^ cells/ml) were cultured following the removal of erythrocytes at for 72 h in CM. To culture purified populations, mononuclear cells were prepared from a Lympholyte M (Cedarlane Labs) gradient, and γδ T cells along with an enriched T cell fraction were purified using a γδ T cell isolation kit (Miltenyi Biotec), according to the manufacturer's instructions. Cells were cultured at 5×10^5^ cells/ml in CM alone or in the presence of ionomycin (500 ng/ml)/PMA (50 ng/ml) or plate bound anti-TCRγδ mAb (UC7-13D5; 100 µg/ml) for 72 h at 37°C/5%CO_2_. Supernatants were also harvested from murine peritoneal macrophages and bovine macrophages (in some instances co-cultured with autologous γδ T cells), which were infected with *B. abortus*. All supernatants were filtered through a 0.4 µm filter and stored at –80°C. Capture ELISA for IL-4, IL-6, IL-10, IL-17, IFN-γ, and TNF-α was used to quantify cytokine levels, as previously described [45], [46], [49].

### Generation of Bovine Monocyte-Derived Macrophages

Whole blood was collected from 6–10 month old Holstein calves into sodium heparin tubes (BD Biosciences). Leukocytes were separated from whole blood using Histopaque-1077 (Sigma-Aldrich) for bovine cells, as previously described [47]. Monocyte-derived macrophages were generated via modification of a previous protocol [50]. PBMCs (1×10^7^/ml) were cultured in CM without antibiotics containing 12.5% autologous serum. Cells were allowed to adhere to tissue culture flasks for 2 h at 37°C/5%CO_2_ when the flasks were agitated. The cells were then cultured at 37°C/5%CO_2_ for 72 h when flasks were agitated again, and the supernatant was aspirated and fresh CM without antibiotics containing 12.5% autologous serum was added. Adherent cells were cultured for an additional 72 h at 37°C/5%CO_2_, when the flasks were again agitated, and the supernatant removed. Adherent cells were then briefly trypsinized and the flasks agitated to dislodge the cells, which were washed in fresh CM without antibiotics containing 10% FBS. Monocyte-derived macrophages were then cultured at 2×10^5^/ml in CM without antibiotics containing 10% FBS overnight in 24 well tissue culture plates prior to infection with *B. abortus*. All experiments were approved by the Montana State University Institutional Animal Care and Use Committee (Bozeman, MT).

### Purification of Bovine T Cell Subsets

Bovine PBMCs were isolated as described above and then incubated with biotinylated mAbs against CD4^+^ T cells (clone CC30) [51] or the TCRγδ (clone GD3.8) [52]. Washed cells were incubated with streptavidin microbeads (Miltenyi). Cells were washed again and positively sorted using magnetic LS columns (Miltenyi) according to manufacturer's instructions. The resulting purity was greater than 95%. Purified T cells were allowed to rest overnight and were resuspended in fresh CM the following day immediately prior to being added to wells containing *B. abortus*-infected macrophages.

### Infection of Bovine Macrophage and γδ T Cell Co-Cultures

Bovine macrophages (prepared as described above) were infected with *B. abortus* in a manner similar to that described previously [5]. Macrophages (2×10^5^/ml) were infected with *B. abortus* (30 bacteria∶macrophage) for 1 hour at 37°C/5% CO_2_. Macrophages were then washed with PBS, fresh CM containing 50 µg/ml gentamicin (Sigma-Aldrich) was added, and cells were incubated for 30 min at 37°C/5% CO_2_. After washing twice, as described above, fresh CM containing gentamicin (2.5 µg/ml) with or without autologous CD4^+^ cells or γδ T cells (2×10^6^/ml prepared as described above,) was added, and cells were incubated 37°C/5% CO_2_. A neutralizing anti-bovine IFN-γ mAb (clone CC302, Serotec, 50 ng/ml: [53]) was also added to some wells. At various time points post-infection, the wells were washed three times, macrophages were lysed with sterile water, and intracellular bacterial burden was determined by serial dilutions of macrophage lysates on BA. The nonadherent T cells were aspirated from the co-culture, centrifuged, and stored in Tri Reagent (Sigma-Aldrich) for RNA extraction. In some instances, after the aspiration of T cells (or CM only) and three washes with PBS, adherent macrophages were lysed in Tri Reagent for RNA extraction. Supernatants were also harvested and 0.4 µm- filtered and stored at -80°C to determine IFN-γ levels using mAbs from ABD Serotec: the capture mAb was clone CC330, and the detecting mAb was clone CC302. IL-8 concentrations were also measured using the anti-human CXCL-8/IL-8 DuoSet Kit from R&D Systems, which cross-reacts with bovine IL-8.

### Reconstitution of Rag-1^−/−^ Mice with Bovine Leukocytes

Rag-1^−/−^ mice were depleted of NK cells via i.p. administration of 0.5 mg of an anti-NK1.1 mAb (clone PK136, BioXCell) on days -2 and day 3 post-infection. One day before infection, PBS, macrophages (5×10^5^/mouse) only or macrophages plus T cells (1×10^7^/mouse) were adoptively transferred into mice intraperitoneally. Macrophages and T cells were purified, as described above. One day later, mice were infected i.p. with 1×10^4^ *B. abortus* 2308. Splenic colonization was determined seven days later.

### RNA Extraction and RT-PCR Analysis

RNA was extracted from cells in Tri Reagent according to manufacturer's guidelines. RNA was then further purified via extraction with an RNeasy Mini Kit (Qiagen). cDNA was generated using the Superscript III First Strand Synthesis System (Invitrogen). Primers for immune-related genes along with β-actin (endogenous control) were designed using the PrimerQuest application from IDTDNA.com and purchased from IDT. Amplicons were visualized under UV illumination on a 2% agarose gel containing GelRed (Biotium).

### Statistical Analysis

The Student *t* test was used to evaluate the differences in colonization, splenic weights, cytokine production, intracellular bacterial burden, and lymphocyte populations when two groups were compared. When more than two groups were compared, an ANOVA followed by Tukey's test was used. A *p* value of <0.05 was considered significant.

## Supporting Information

Figure S1 **B. abortus infection does not induce IL-17 or IFN-γ production by γδ T cells.** **A.** Splenocytes from naïve or *B. abortus*-infected mice (7 dpi) were stimulated overnight with PMA/Ionomycin and brefeldin A was added for the last 3 h of culture. Following surface staining, cells were permeabilized and stained for intracellular IL-17 or IFN-γ. Top panel, the proportion of IL-17 producing γδ T cells was determined following gating on lymphocytes. Second panel from top, cells were gated on CD4^+^ (CD3^+^) T cells and assayed for IL-17 production. Third panel from top, cells were gated on γδ T cells (CD3^+^/TCR γδ^+^) and assayed for IFN-γ production. Bottom panel, cells were gated on CD4^+^ (CD3^+^) T cells and assayed for IFN-γ production. Depicted is the mean ± SD of 5 mice/group and is representative of two independent experiments. **B.** γδ T cells were sorted from naïve or *B. abortus*-infected (7 dpi) mice and stimulated for 72 h with PMA/Ionomycin. Cytokine levels in supernatant were determined by ELISA. Depicted is the mean ± SD of triplicate wells. *P<0.05 versus cytokine production by γδ T cells from naïve mice.
(TIF)

Figure S2 **IL-8 production is augmented by γδ T cells when co-cultured with bovine macrophage during infection with** ***B. abortus*** **.** IL-8 concentrations were measured by ELISA in supernatants from *B. abortus*-infected bovine macrophages cultured with or without autologous T cells at various time points after infection. Data depict the mean ± S.D. of triplicate measurements/group. *P<0.05 versus wells containing macrophages only.
(TIF)
