# Supplementary figures and images for: Murine and Bovine γδ T Cells Enhance Innate Immunity against Brucella abortus Infections
Source: PLoS One. 2011 Jul 12;6(7):e21978. doi: 10.1371/journal.pone.0021978 (PMC3134454; doi:10.1371/journal.pone.0021978)

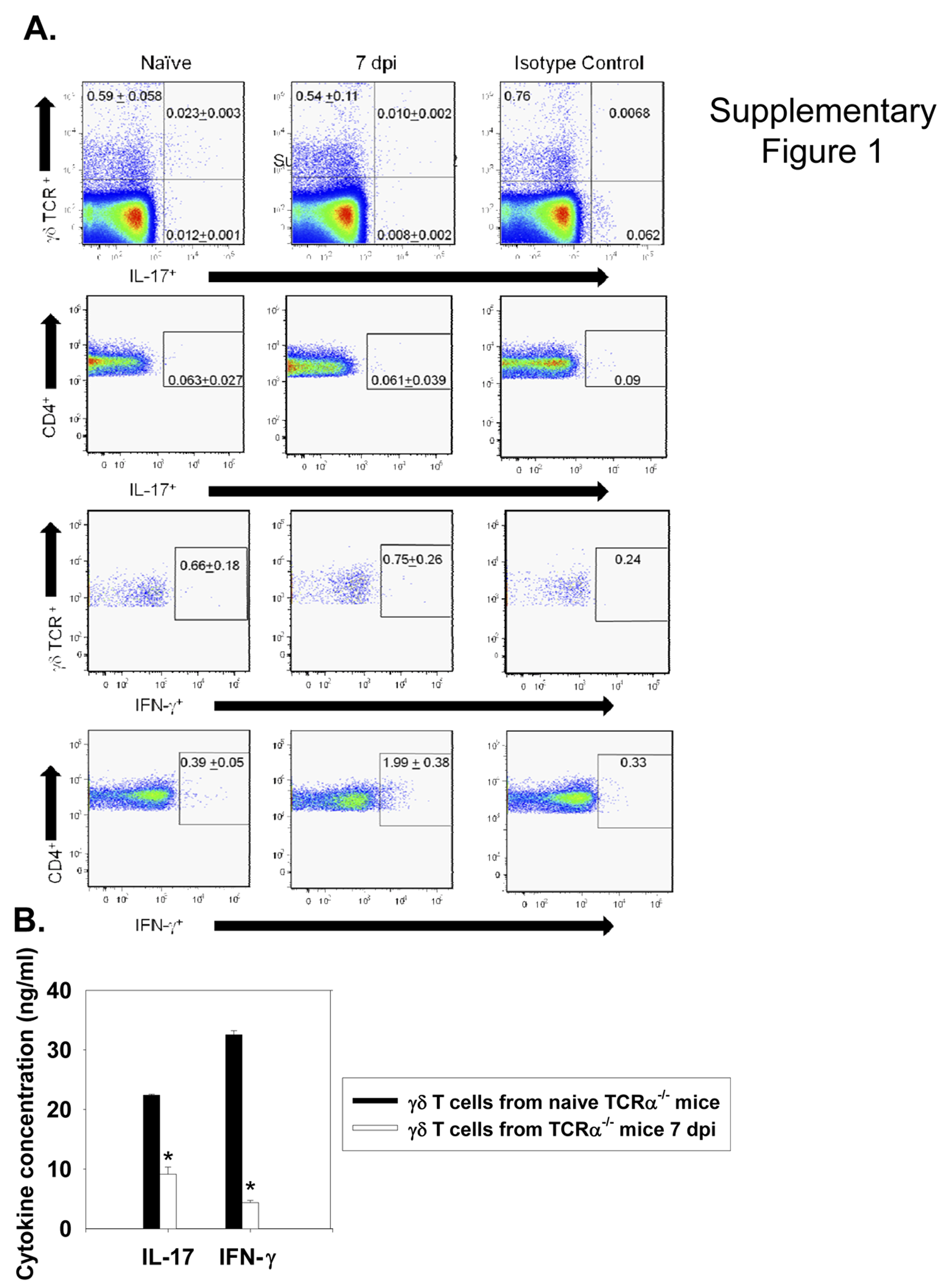

Supplement: Figure S1 — B. abortus infection does not induce IL-17 or IFN-γ production by γδ T cells. A. Splenocytes from naïve or B. abortus-infected mice (7 dpi) were stimulated overnight with PMA/Ionomycin and brefeldin A was added for the last 3 h of culture. Following surface staining, cells were permeabilized and stained for intracellular IL-17 or IFN-γ. Top panel, the proportion of IL-17 producing γδ T cells was determined following gating on lymphocytes. Second panel from top, cells were gated on CD4+ (CD3+) T cells and assayed for IL-17 production. Third panel from top, cells were gated on γδ T cells (CD3+/TCR γδ+) and assayed for IFN-γ production. Bottom panel, cells were gated on CD4+ (CD3+) T cells and assayed for IFN-γ production. Depicted is the mean ± SD of 5 mice/group and is representative of two independent experiments. B. γδ T cells were sorted from naïve or B. abortus-infected (7 dpi) mice and stimulated for 72 h with PMA/Ionomycin. Cytokine levels in supernatant were determined by ELISA. Depicted is the mean ± SD of triplicate wells. *P<0.05 versus cytokine production by γδ T cells from naïve mice. (TIF) [file pone.0021978.s001.tif]

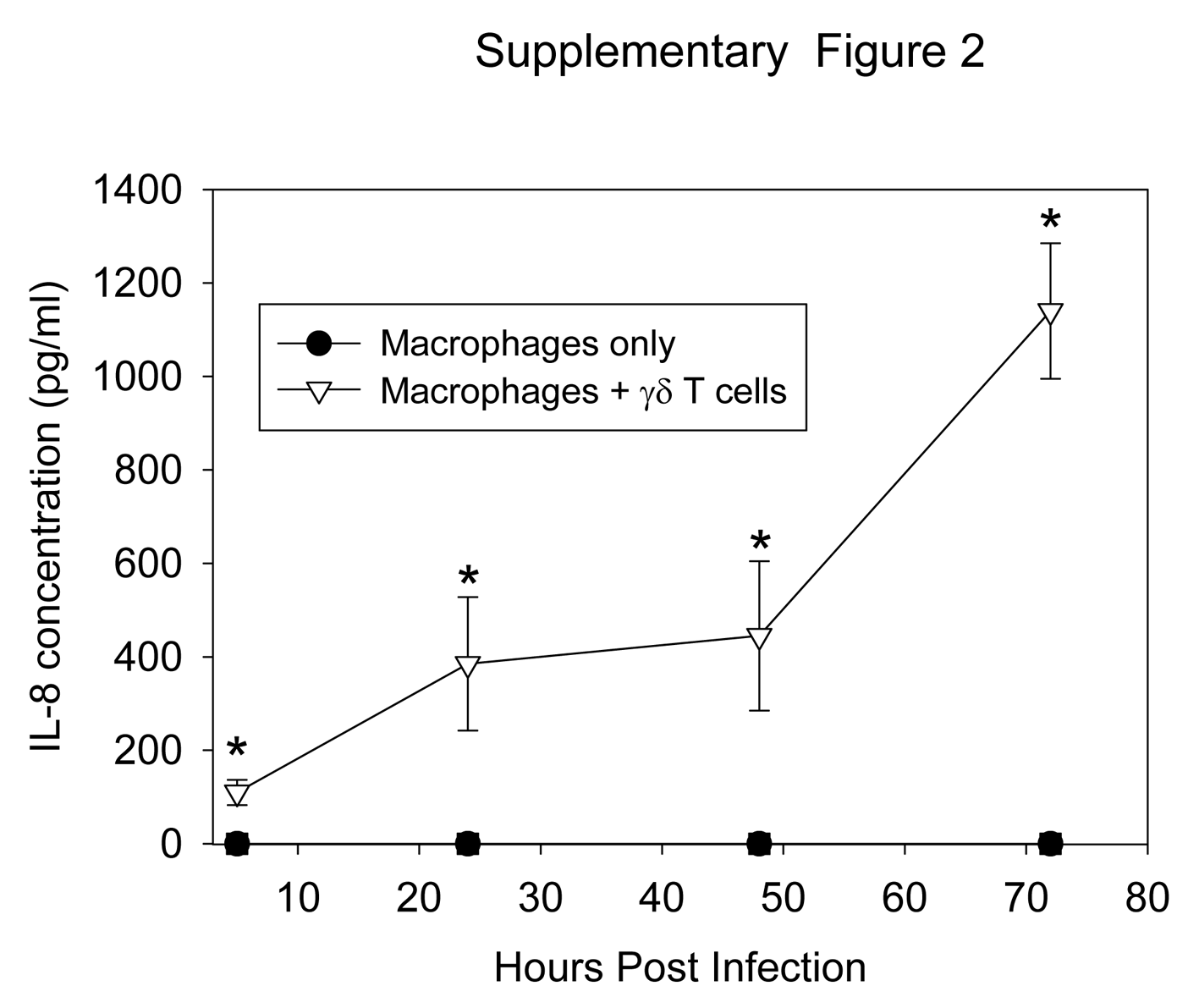

Supplement: Figure S2 — IL-8 production is augmented by γδ T cells when co-cultured with bovine macrophage during infection with B. abortus . IL-8 concentrations were measured by ELISA in supernatants from B. abortus-infected bovine macrophages cultured with or without autologous T cells at various time points after infection. Data depict the mean ± S.D. of triplicate measurements/group. *P<0.05 versus wells containing macrophages only. (TIF) [file pone.0021978.s002.tif]
